# Suggestions to ameliorate the inequity in urban/rural allocation of healthcare resources in China

**DOI:** 10.1186/1475-9276-13-34

**Published:** 2014-05-01

**Authors:** Yiyi Chen, Zhou Yin, Qiong Xie

**Affiliations:** 1 The First Affiliated Hospital, College of Medicine, Zhejiang University, 79 Qing Chun Road, Shang Cheng District, Hangzhou 310003, Zhejiang Province, China; 2 The Second Affiliated Hospital of Zhejiang Chinese Medical University, 318 Chao Wang Road, Gon Shu District, Hangzhou 310014, Zhejiang Province, China

**Keywords:** Urban–rural gap, Healthcare resources, Allocation, Inequity

## Abstract

The imbalance in the allocation in healthcare resources between urban and rural areas has become a main focus of the recent medical reforms adopted in China. However, systematic analysis has identified wide differences in the allocation of healthcare resources between urban and rural areas, including healthcare expenditures and the number of healthcare facilities, available beds, and personnel. Therefore, the aim of this report was to identify ethical considerations in current governmental policies to rectify existing problems in the distribution of healthcare resources. Our findings indicate that the inequality in the distribution of healthcare resources does not adhere to ethical standards and the policies are flawed because they give rise to differences in the availability of medical care to urban and rural communities. To optimize the allocation of medical healthcare resources, countermeasures are proposed to formulate policies to urge the flow of public healthcare resources to rural areas, strengthen the responsibilities of both governmental and public financial investments, increase the construction of public healthcare facilities in rural areas, promote the quality of healthcare resources, adjust resource allocations to rural public healthcare facilities, and improve resource utilization efficiency by establishing two-way referral mechanisms.

## Introduction

Financial, material, and human resources are the foundation of the healthcare services provided by the Ministry of Health of the People’s Republic of China to ensure the accessibility of quality healthcare services. However, how to allocate healthcare resources is not only an economic issue constrained by the level of economic development, but also embodies certain ethical issues. Furthermore, the distribution of financial, material, and human resources should meet the requirements of contemporary medical ethics as such decisions directly impact the future direction of development of the national health care system, promotion of medical science technology, and methods to monitor the general health of the population.

Appropriate healthcare standards and meeting ethical considerations of rural populations have emerged as key concerns in the national discussion on disparities in the availability of healthcare services [1-9]. The launch of new reforms has opened a new stage of healthcare mandates and also brings both opportunities and challenges to achieve an equilibrium in the allocation of healthcare resources. Therefore, the aim of this study was to analyze current problems in the allocation of public healthcare resources in rural areas of China and to describe the disparity in healthcare funding, insufficiently funded healthcare facilities, shortages in human resources, and the structural imbalance in resource allocation. In addition to the unequal distribution of healthcare resources between urban and rural areas, the ethical issues regarding an urban-rural dual structure, the urban-rural income gap, and the insufficient attention from local government on public health are also addressed.

## Methods

Data used for this analysis were obtained mainly from the China Health Statistical Yearbook. The study was approved by the Ethics Committee at the First Affiliated Hospital, College of Medicine, Zhejiang University.

## Differences between urban and rural areas

### (1) Medical and healthcare expenses

**Figure 1:**
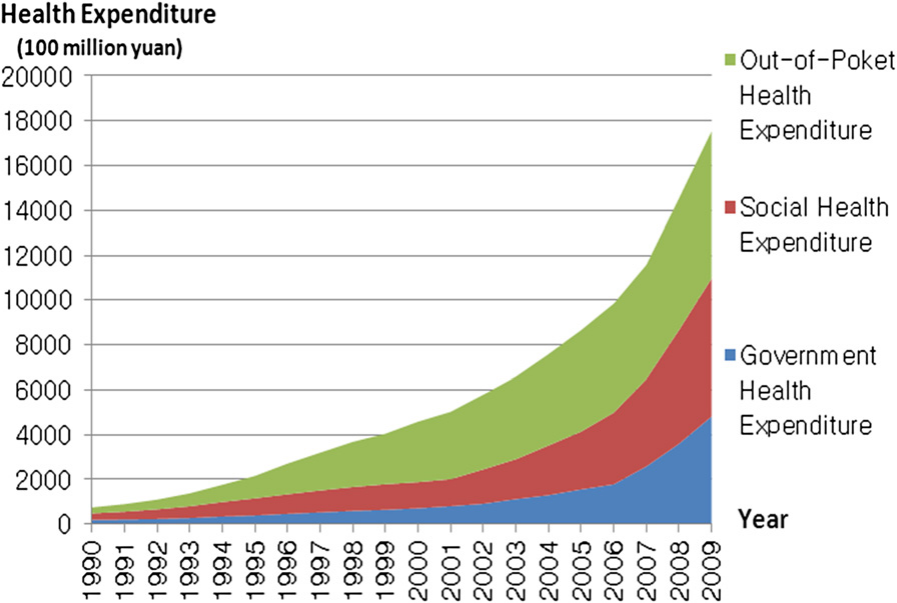
Yearly growth in health expenditures.

From 1900 to 2009, the total health expenditure (THE) in China has increased from 74.74 billion yuan (¥) to 1.75419 trillion and, against this background, the gap between urban and rural areas has continued to grow, which is reflected in the ratios of healthcare expenditures between these areas over the past two decades (1990, 53.0% and 47.0%; 1995, 57.5% and 42.5%; 2005, 72.6% and 27.4%; and 2008, 77.4% and 22.6%, respectively; Figure 1).

As shown by the two sets of data presented in Table 1, the per capita healthcare expenditure between urban and rural areas continues to vary tremendously: 1990, 158.8 ¥ and 38.8 ¥ (ratio, 4.09:1); 1995, 401.3 ¥ and 112.9 ¥ (ratio, 3.55:1); 2000, 813.0 ¥ and 214.9 ¥ (ratio, 3.78:1); 2005, 1126.4 ¥ and 315.8 ¥ (ratio, 3.57:1); and 2009, 2176.6 ¥ and 562.0 ¥ (ratio, 3.87:1), respectively. A simple interpretation of these data suggest that if one quarter of urban health costs were shifted to rural areas, the per capita health expenditure in rural areas would double, thereby ameliorating the lack of medical services, while greatly enhancing the effectiveness of healthcare delivery and patient satisfaction, and meeting service demands of the population.

**Table 1:** The growth of total expenditure on health (TEH), total health expenditure as% of GDP (THE as% of GDP) and Per Capita Health Expenditure (Per Capita THE)

**Indicator**	**2000**	**2005**	**2007**	**2008**	**2009**
**TEH (100 million ¥)**	4586.6	8659.9	11573.9	14535.4	17541.9
**THE as % of GDP (%)**	4.62	4.68	4.35	4.63	5.15
**Per capita THE (¥)**	361.9	662.3	875.9	1094.5	1314.3
**Urban**	813.0	1126.4	1516.3	1861.8	2176.6
**Rural**	214.9	315.8	358.1	455.2	562.0

### (2) Number of healthcare institutions and beds

The number of the healthcare clinics located in towns and townships declined in the decade from 2000 to 2010 from 49229 to 37836, respectively, and the number of healthcare clinics located in villages has rapidly decreased from 709,458 in 2000 to 514,920 in 2003, but had since grown to 648,424 by 2010.

Between 1980 and 2006, the number of beds in both hospitals and clinics in China increased from 2.184 million to 3.271 million, which including a 249.83% increase in cities from 903,000 to 2.256 million, while decreasing by 79.08% in rural areas from 1.2817 million to 1.103 million.

### (3) Number of healthcare professionals

From 2000 to 2010, the average number of healthcare technicians available in rural healthcare centers has steadily increased from 23.0 in 2002 to 25.9 in 2007 and to 30.4 in 2010. Furthermore, the number of doctors per village and per 1000 rural population has gradually increased over this period, but is still far below the national average. By the end of 2010, there were 5,876,158 licensed healthcare technicians nationally: 2,954,913 in urban facilities and 2,911,245 in rural facilities. Nationally, there are 4.37 health technicians per 1000 population, but this ratio varies greatly between urban and rural areas (7.62 and 3.04, respectively). In addition, the quality of healthcare services in rural areas was significantly lower than that in urban areas, as most healthcare practitioners did not have a bachelor’s degree and many were uncredited altogether.

### (4) Health status of the population

As a result of the inequity in the allocation of healthcare resources, the health of urban and rural populations varied significantly. For example, the maternal mortality rate in rural areas was two- to three-fold greater than that in cities and towns from 1995 to 2005; however, this trend gradually narrowed after 2006 and was resolved by 2010 (Table 2). At the same time, vast differences still existed in many aspects of healthcare availability, as demonstrated by the neonatal mortality rate, infant mortality rate, and mortality rate of children aged >5 years between urban and rural areas (4.1% vs. 10.0%, 5.8% vs. 16.1%, and 7.3% vs. 20.1%, respectively; Table 3), according to the 2011 edition of the Chinese Health Statistical Digest.

**Table 2:** Maternal mortality rate (per 100,000 live births)

**Year**	**Total**	**Urban**	**Rural**
**1990**	88.9	45.9	112.5
**1995**	61.9	39.2	76.0
**2000**	53.0	29.3	69.6
**2005**	47.7	25.0	53.8
**2006**	41.1	24.8	45.5
**2007**	36.6	25.2	41.3
**2008**	34.2	29.2	36.1
**2009**	31.9	26.6	34.0
**2010**	30.0	29.7	30.1

**Table 3:** Mortality rate of newborns, infants, and children (per 1000 live births)

**Indicator**	**2000**	**2005**	**2008**	**2009**	**2010**
**Total**					
**Newborn mortality**	22.8	13.2	10.2	9.0	8.3
**Infant mortality**	32.2	19.0	14.9	13.8	13.1
**Mortality of children >5 years**	39.7	22.5	18.5	17.2	16.4
**Urban**					
**Newborn mortality**	9.5	7.5	5.0	4.5	4.1
**Infant mortality**	11.8	9.1	6.5	6.2	5.8
**Mortality of children >5 years**	13.8	10.7	7.9	7.6	7.3
**Rural**					
**Newborn mortality**	25.8	14.7	12.3	10.8	10.0
**Infant mortality**	37.0	21.6	18.4	17.0	16.1
**Mortality of children >5 years**	45.7	25.7	22.7	21.1	20.1

## Key issues regarding allocation of healthcare resources

### (1) Greater emphasis on efficiency than fairness

Fairness and efficiency are the two most basic principles of economic activity and also act as a scale to assess basic moral values in the process of allocating urban-rural healthcare resources. For example, the prevalence of some diseases, such as chronic metabolic diseases, chronic mental illnesses and so on, once perceived as “city problems,” have been increasingly diagnosed among rural populations, mirroring those of urban populations [10-12]. At the same time, compared to urban populations, those residing in rural areas reportedly are at a higher risk of other chronic illnesses, such as cancer, heart disease, and arthritis [13]. However, due to history and economic reasons, under the guidance of the “efficiency first and equity considered” policy, and the nature of the established market economy to obtain profit, the government has failed to address the inadequacies in efficiency and have neglected the ethical standard of fairness, resulting in a disparity in resources relegated to cities. One possible reason for this discrepancy in the promotion of a medical security system in rural communities may be that rural governments prioritized the agricultural industry for quite a long time and inadequately invested in basic public infrastructures regarding social security.

### (2) Lack of invest in the rural medical

With the background of economic reforms and social transformation, the Chinese government has given too much attention to economic growth, while largely ignoring social services, especially healthcare. All levels of government rely on economic indicators, such as growth rates, gross domestic product (GDP), and financial income growth, and integrate these indicators in evaluation indices of achievements and promotion, but tend to neglect social indicators relative to individual livelihood.

### (3) Blind expansion of large-scale hospitals in urban areas

Some large and medium-sized cities in China with high population concentrations have formed unique medical markets since undergoing economic development to meet the needs of populations with greater demands and the ability to pay for healthcare services. Moreover, in response to the vast number of Medicare patients, local governments have blindly expanded the number of large medical institutions and facilities to quicken the availability of healthcare resources in cities with greater population densities. Conversely, it is very difficult for rural areas to expand healthcare resources and purchase high-tech equipment due to the relatively poorer economic conditions, lack of government investment, and low payment capacity. Above all, with the combined effect of the tendency of market profit and differences in economic conditions between urban and rural areas, the gap in allocation of medical resources between urban and rural areas has increased.

### (4) The lack of ethical considerations in policy implementation

In 2003, The Chinese government began to implement cooperative medical systems in some provincial rural areas and have strengthened these efforts by adjusting policies in 2006 and established the basic goal of covering all rural residents by 2010 to resolve primary healthcare inadequacies faced by farmers. Nevertheless, during the process of implementing these policies, existing issues, such as poor execution of these policies by some local governments, as well as the phenomenon of fraud, poor management, and inappropriate funding has disillusioned those living in rural communities to some extent and has adversely affected the stable implementation of newly formed rural cooperative healthcare systems.

## Main measures to optimize the configuration

### (1) Related policies to guide the flow of public healthcare resources to rural areas

The allocation of public healthcare resources plays a core role in healthcare systems and is also crucial to reform and developmental processes. However, the interests of the state as well as the physical and mental healthcare needs of the population must be considered when implementing healthcare policies and allocating resources. Therefore, policies to achieve equality in the allocation of public healthcare resources should be instituted. Moreover, fairness regarding value distribution and ethical considerations in the allocation of healthcare resources should be upheld to ensure fair distribution. A primary goal of the national healthcare system is to ensure and protect basic human rights, thus the government must develop an effective system to supply healthcare services, while balancing demands, fairness, and availability of benefits. Also, it is necessary for the government to assume the primary responsibility of investment in and management of medical services in rural areas and to insist on public responsibility and fairness to promote the transfer of state finances to further develop rural healthcare services.

### (2) Expansion of the construction of healthcare centers in townships

The rural healthcare system is a social project. As an important part of the three-link healthcare network in rural areas, it is necessary to identify the attributes, choose locations, define responsibilities, and consider the scale of the township governmental body charged with the important tasks of epidemic prevention and healthcare administration to discern the difficulty and costs associated with the assessment of healthcare services in rural areas. The following factors should be considered regarding the construction of healthcare centers in townships: (i) attributes: township healthcare centers are non-profit medical institutions servicing at least one village while maintaining governmental standards; (ii) location: township healthcare centers should adhere to accepted management practices of the county and township, be independently managed, assume the sole responsibility for profits or losses, and establish cooperative relationships with county-level hospitals and village clinics, but not competitive relationships; (iii) responsibility: as an integral institution responsible for prevention of epidemics, the township healthcare center is responsible for implementation of local healthcare policies, the activities of medical organizations, and training of healthcare workers; and (iv) scale: it is necessary to ensure the scale of hospitals and number of personnel to accommodate the local population in the service area.

To increase input and set up mechanisms to finance rural healthcare services, the government should play a key role in the channeling of investments in the form of subsidies to rural hospitals and strictly implement funding for preventive medicine programs, construction of basic medical facilities, and regular wages for healthcare workers. Secondly, new social financing channels must be explored to develop collaborations with businesses to develop multi-channel financing arrangements based on public needs. Thirdly, independent investments in township healthcare centers should be encouraged through multi-pronged methods, such as the use of rewards instead of investment and preferential policies.

To attract and train new talent, improvements in healthcare systems in rural areas are needed. Local governments at various levels should institute effective measures to encourage recruitment of highly educated healthcare technicians and managerial personnel to township hospitals [14,15]. Residency programs with various shift systems have been designed with the intent to change the entire structure of rural medical human resource to retain doctors and bring new healthcare workers to township healthcare centers that currently lack trained medical professionals to meet the basic needs of rural areas and improve the availability of economic and social benefits.

### (3) Establish and perfect two-way referral mechanisms

Most residents who live in rural areas must travel to receive primary healthcare services, as elsewhere in the developing world [16]. Rural residents travel longer distances and have more difficulty in receiving referrals for medical procedures than urban residents, which pose significant barriers to care, particularly for the elderly and children [17,18]. Therefore, a dual referral system is an integrated component in primary healthcare services in rural communities. The practical significance of the dual referral system is very obvious, as it is conductive to the reallocation of healthcare resources to improve the effective utilization of resources and reduce the burden of medical patients to receive care at rural primary health institutions and facilitate disadvantaged groups living in rural areas to consult a doctor. Healthcare administrators should expeditiously formulate a practical program to identify indications, principles, standards, and processes for referral to higher care-level hospitals. At the same time, administrators should also establish relevant organizations, support systems, rural health information exchange platforms, systems to foster interactions between medical institutions at all levels, and broaden cooperation channels to promote the sustainable development of a dual referral system. To achieve this goal, major city hospitals should consider taking over the operation of hospitals in small towns or townships as branches by means of mergers or rent depending on the specialties of the facilities and management advantages, which constitutes a primary network to provide medical services to rural areas. New medical service groups are not only important to straighten out the distribution of medical resources to towns, but also promote the effective flow of quality medical resources between urban and rural areas.

## Discussion

In many countries, the allocation of healthcare resources may be unreasonable. For rural residents, limited economic resources [4,19], limited availability and accessibility to healthcare services [19-21], cultural and personal values, overlapping professional–patient relationships and caregiver stress [4,7,22-25], as well as geographical and climate factors affecting travel have been identified as the main barriers to access of healthcare services [4,7,22]. Each of these factors contributes to the main healthcare issue that residents of rural areas are more likely to have chronic health problems and poorer health status than their urban counterparts [3,4,26-28].

Healthcare is considered a social welfare service, thus the government is required to improve and maintain equal access of healthcare services to both urban and rural residents. Generally speaking, the reasonable allocation of healthcare resources can be thought of as an “equilateral triangle,” which refers to allocation of healthcare resources that flow into rural areas with greater populations and greater healthcare needs. As a matter of fact, the allocation of healthcare resources in China is actually an “inverted triangular.” Against the imbalance in the allocation of healthcare resources to rural areas, the primary problems faced by healthcare management include the use of existing healthcare resources with maximum efficiency and promotion of the rural medical work according to the principles of balancing equity and efficiency, improving accessibility and overall benefits of health services, and disease prevention and control.

Presently, there are no specific standards to evaluate fairness in the allocation of healthcare services by the national health system. Nevertheless, the guarantee that all people enjoy basic healthcare services is the key factor to evaluate healthcare systems. Although the constitution of the World Health Organization (WHO) states that “the enjoyment of the highest standard of health is one of the fundamental rights of every human being, without distinction of race, religion, political belief, economic and social condition,” the inequity in allocation of healthcare resources is problematic worldwide, not just a unique phenomenon in China. In light of these goals, many countries have formulated ambitious healthcare reform plans. The most recent healthcare reform policy in China (released in April 2009) explicitly recommends the promotion of healthcare resource utilization and increased construction of healthcare facilities in rural areas to promote community health. For now, healthcare reform both in the US and China are receiving resistance due to contradictions in economic policies. The current problems faced by healthcare reform in the US are external obstacles from various groups with financial interests, while the main problem faced by the basic healthcare system in China is the system itself. In the future, the direction of healthcare reform in China will tend to be management-oriented marketization, which aims to reverse the current inequity in healthcare resource allocation and extreme treatment options available to patients, and to consider the effectiveness of mechanisms to grade healthcare services to ensure a reasonable flow of healthcare resources. In China, the main obstacle to convert the current system of over-administered healthcare into a social mechanism requires adjustments to existing structures; however, conflicts of interest may become major obstacles. To promote healthcare reform, the government should perform four functions: act as an insurer to establish universal coverage through a medical security system; act as a purchaser to limit the rising costs of medical services; act as a resource allocator to set up an efficient primary healthcare system; and act in a supervisory capacity to control market failures of medical services.

## Competing interests

The authors declare that they have no competing interests.

## Authors’ contributions

YC and QX formulated the research concept and design. All authors were involved in data collection. YC and ZY wrote the manuscript and QX read and commented on the paper. The final version submitted for publication was read and approved by all authors.
